# Public thoughts on incentivizing COVID-19 vaccine uptake in the United States: testing hostile media bias with user-generated comments

**DOI:** 10.3389/fsoc.2023.1041454

**Published:** 2023-10-31

**Authors:** Sherice Gearhart, Ioana A. Coman, Alexander Moe, Sydney E. Brammer

**Affiliations:** ^1^Department of Public Relations and Strategic Management Communication, College of Media and Communication, Texas Tech University, Lubbock, TX, United States; ^2^The State University of New York (SUNY) Brockport, Brockport, NY, United States

**Keywords:** hostile media bias, Facebook, news, comments, perception, COVID-19

## Abstract

Facebook is the most popular social media platform and often used by news organizations to distribute content to broad audiences. Features of this online news environment, especially user-generated comments shown to news consumers, have the potential to induce audience perceptions of hostile media bias. This study furthers investigation into the influence of exposure to Facebook comments and news topics on consumers. Using a sample of U.S. adult Facebook users (*N* = 1,274), this work utilized a 2 (likeminded comments or disagreeable comments) × 2 (story topic of requiring COVID-19 vaccines to receive a monetary bonus or maintain employment) between-subjects experimental design. While controlling for the influence of partisanship, this work further proves that features of the Facebook environment uniquely influence news audience perceptions of neutral news content. Specifically, findings indicate that news story topic can influence perceptions of bias. Further, topic and comment exposure interacted, demonstrating the intensity of story topic and likeminded comments enhance hostile media perceptions.

## Introduction

Deployment of the COVID-19 vaccine across the United States (U.S.) began in early 2021, with an initial focus on older adults and vulnerable populations. By June 2021, the vaccine became available to all adults and children aged 12 and above. According to public health experts, vaccination remains the best way to end the global COVID-19 pandemic, especially as society attempts to stay open. However, while public health experts and public figures encouraged vaccination, segments of the U.S. population remained skeptical. Just as mask-wearing was divisive, the subsequent vaccines were further politicized (Albrecht, 2022; Bolsen and Palm, 2022).

Media outlets and public service campaigns joined medical professionals' efforts to increase vaccine uptake. Non-profit organizations such as the Advertising Council launched multi-million-dollar campaigns targeting a wide variety of communities to help them overcome the hesitancy and skepticism that characterized the vaccine conversation (Montgomery, 2021). These carefully tailored pro-vaccination campaigns made their way into mainstream news and social media, successfully gaining traction among wider audiences. One of a journalist's primary functions is to equip citizens with the information they need to make critical decisions. During the COVID-19 pandemic this included information about this new virus and how to prevent it (i.e., the COVID-19 vaccine; Dean, 2021).

In the United States, 53% of adults get their news from social media; online news was and continues to be a major source of COVID-19 and vaccine information (Shearer, 2021). However, the online news environment created by social media platforms like Facebook introduces the influence of other users into the picture, shaping the transfer of information in a new way. For example, a Facebook user is subject to viewing a teaser posted by news outlets to promote a particular news story while simultaneously encountering comments posted by other users *before* viewing the article.

As a result of technological changes, news outlets increasingly use social media for news dissemination and news consumers are exposed to comments posted by others before they read a news article. Therefore, this work is theoretically guided by the hostile media bias. The first aim of this study is to experimentally test whether comments, appearing below a Facebook post promoting a news story, influence perceptions of bias. In addition, this work aims to test whether the influence of comments vary across news stories related to different tactics used to encourage COVID-19 vaccines. Results provide further evidence of the hostile media phenomenon in social media contexts and expand the theoretical knowledge and breadth of application.

## Literature review

### Theoretical foundation: hostile media bias

Hostile media bias is a phenomenon that occurs when audiences perceive media coverage as being biased against their opinion (Perloff, 2015). In their seminal experimental investigation into the theory, Vallone et al. (1985) exposed participants to a neutral news segment depicting the 1982 invasion of Lebanon by Israel to establish hostile media bias as an ongoing phenomenon. Results indicated that participants who harbored strong partisan views or were highly invested in an issue perceived media coverage as biased against their own view or sentiment, a finding that subsequent hostile media bias researchers later replicated (e.g., Vallone et al., 1985; Gunther, 1992; Dalton et al., 1998). While this early work was instrumental in establishing the hostile media phenomenon, the focus only on strong partisans may been seen as a shortcoming.

A growing body of empirical work testing hostile media bias has observed a variety of mediums, including newspaper, television, and social media, across a wide range of content with the added notion that sources (e.g., channels or outlets) may impact perceptions of hostile media bias (Gearhart et al., 2020). Past researchers have attempted to determine what factors can contribute to hostile media bias as it may influence other important communication behaviors such as audiences' explorations of social alienation and political dialogue (Tsfati, 2007; Barnridge and Rojas, 2014; Perloff, 2015). Identified factors have included the likelihood to assimilate, accept face-value information, or scrutinize information that opposes partisan positions, along with common social identity precursors (e.g., Lord et al., 1979; Perloff, 1989; Giner-Sorolla and Chaiken, 1994). For example, Tsfati (2007) found that minority perceptions of media bias might heighten minority opinion holders' feelings of alienation or social exclusion. Further, Barnridge and Rojas (2014) found perceived bias in media content “makes people attempt to ‘correct' perceived ‘wrongs' by voicing their own opinions in the public sphere” (p. 135).

Studies on hostile media bias have turned their attention toward online news stories from both mainstream outlets and blogs and found consistent results (e.g., Gunther and Liebhart, 2006; Kim, 2015). Perloff (2015) claims a gap in scholarly understanding of how hostile media bias occurs via online platforms that remain limited or nascent at best. Specifically, there is an urgent need for research focusing on user-generated comments paired with online news. User comments may influence perceptions due to the ability of third-party audience members to shape others' perceptions of a news story or its editorial frame and may ultimately contribute to perceptions of an implied lean to the story depending on the audience's pre-conceived sentiments (Gearhart et al., 2021). Moreover, studies found that user comments may exaggerate elements that appear within news segments and may further guide certain cognitive processes (Lee and Tandoc, 2017). Individuals who perceive the media as hostile to their own views will attempt to resolve this perceived hostility by discussing political issues more often, including discussion in comment sections under a news story (Barnridge and Rojas, 2014). As such, user-generated comments are an important and essential consideration of this current study, providing a unique avenue for the extension of hostile media bias research.

Building on these observations, it is necessary to examine whether elements of online news consumption platforms can further influence hostile media perceptions. Facebook, one of the most popular social media platforms used by nearly 70% of American adults, is a popular place for users to share and consume news content (Auxier and Anderson, 2021). Elements of this platform, including user-generated comments, are displayed to news consumers and have the potential to differently influence perceptions of hostile media bias (Tsfati, 2007; Barnridge and Rojas, 2014; Perloff, 2015; Gearhart et al., 2020, 2021). Further, comments may have varying influence of comments across politically related, controversial issue topics. Therefore, the current study examines whether encounters with user comments influence perceptions of news content across topics.

### Journalistic objectivity: perceptions of bias and ethical values

One other important aspect to consider when examining hostile media bias influenced by user-generated comments is the role of journalistic objectivity. In an effort to maintain fairness and civility, the U. S. Federal Communications Commission (F.C.C.) concluded in 1949 that the duty of broadcast licenses was to cover controversial issues in a fair and balanced manner, also known as *The Fairness Doctrine* (Ruane, 2011). This duty required that broadcasters “devote a reasonable portion of broadcast time to the discussion and consideration of controversial issues of public importance” (Caldera, 2020, p. 11), making available for the expression of opposing perspectives that stem from responsible elements within controversial issues. Abolished in 1987 by the F.C.C., *The Fairness Doctrine* was perceived as hindering the types of democratic debate it was intended to promote (Hershey, 1987). However, it was not formally rescinded until 2011 (Matthews, 2011). The decision to abolish *The Fairness Doctrine* has been attributed to increases in conservative talk radio, which gained popularity during the 1980s and 1990s. Among other forms of journalistic activity, political talk radio was rationalized in 1987 to fall under first amendment protections of speech, despite mostly advocating for a certain partisan viewpoint (Hagey, 2011). This discussion is relevant to today's social media environment, which is driven by capitalistic forces and allows controversial and problematic discourse to accompany legitimate news content, potentially influencing audiences. This threatens perceptions of journalistic objectivity, which has long been the gold standard.

Furthermore, especially in times of crisis, news outlets and journalists are expected to go beyond simply covering the news to help the public make sense of what is going on, solve or better the situation, and even cope (Grusin and Utt, 2005; Bressers and Hume, 2012). Bias, a dimension of credibility, and credibility itself have long been subjects of research (e.g., Rouner et al., 1999; Fico et al., 2004; Gil de Zúñiga et al., 2018). For example, in a vaccine context, Gunther et al. (2012) found anti-vaccination partisans perceived pro-vaccine content as more unfavorably biased. Moreover, research has shown that different factors can affect the public's perceptions of bias in a news story, from the story's internal elements such as structure (Fico et al., 2004) to external elements such as the comments accompanying the news article (Weber et al., 2019), or social media news posts (Kümpel and Unkel, 2020).

### Perceptions of ethical values in journalism

Closely related to journalistic objectivity, another fundamental aspect of journalism is the assumption that each journalist adheres to personal values, attitudes, and philosophical principles that affect how the profession is carried out and ultimately perceived. Journalists also see themselves as governed by outside social influences, such as professional norms, the law, and intrinsic motivations (Voakes, 1997). Journalistic codes of ethics come into play when ethical dilemmas arise. Yet, it remains the responsibility of each journalist to abide by these guidelines at an individual level by drawing their own lines between what they consider to be ethical and not (Holt, 2012). However, it is not enough for journalists alone to be ethical. The way publics perceive the ethics of news media is also very important. For example, Culver and Lee (2019) found that liberals were more likely to perceive news media as ethical than conservatives and, consequently, trusted media more than their conservative counterparts. Even more critically, they found that audiences' participation in the news (e.g., sharing the story with a friend or commenting on a story) was positively connected to perceptions of ethical performance. More recently, according to a Pew Research Center study, Americans ranked journalists' ethical standards below doctors, police, and clergy (Gottfried et al., 2020). Moreover, Democrats were found “far more likely” than Republicans to believe journalists have high or very high ethical standards (Gottfried et al., 2020).

### Journalism and the struggle to maintain an unbiased perception

While journalistic objectivity and ethical values remain critical to the profession, modern journalism continues to struggle with its audience's migration to online spaces and its potential effect on their perceptions. As such, a two-pronged ethical tension has reared its head. While traditional journalism values accuracy, verification, balance, gatekeeping, and impartiality, online journalism seeks to emphasize immediacy, transparency, partiality, and post-publication corrections when or if needed. However, fake news, disinformation, political propaganda, and other creative forms of masking content as “Journalism” remain a direct threat to democracy as it unveils new frontiers for free-speech advocates, among them policymakers, pundits, and professional journalists (Wagner and Boczkowski, 2019). Furthermore, digital technology and ruthless politics alongside commercial exploitation of mass disseminated content, whether in audio, print, broadcast, or digital formats, has contributed to concerns pertaining to bias, ethics, and the foundations of classical democratic procedures for the foreseeable future (White, 2017). Finally, according to Wagner and Boczkowski (2019), perceptions of the media landscape include: (a) carrying a negative connotation of the overall perception of the quality of news reporting; (b) ever-existing distrust of news circulation, specifically as it appears on social media; and (c) an overall concern about the effects of these trends mainly on the perceived information habits of others. Considering these historical backgrounds and current concerns, this study aims to discern how ethical participants perceive stories to be.

### Issue topics: COVID-19 vaccine requirements

Controversial news story topics are ideal for applying the hostile media bias phenomenon in experimental settings. Thus, COVID-19 vaccination is a suitable topic for application and empirical testing. In particular, the implementation of tactics used to encourage vaccine uptake in the workplace have been and continue to be highly contested in the U.S. Since not all Americans took the free and widely available vaccines, private employers took additional measures to encourage the COVID-19 vaccine. For instance, some employers in the U.S. began offering incentives for recipients of the COVID-19 vaccination (Terrell, 2021), while others started to mandate that employees get vaccinated to remain in their positions (Messenger, 2021). However, both practices were deemed controversial in different ways by some publics. Thus, the two types of vaccine requirements (i.e., requiring COVID-19 vaccination to receive a monetary bonus or requiring a COVID-19 vaccination to keep one's job) are used in the current study as controversial topics suitable for testing the hostile media phenomenon.

The controversial nature of news content about COVID-19 vaccination requirements is suitable for testing the hostile media phenomenon. Furthermore, the review above demonstrates how the features of Facebook might inherently expose news consumers to factors that may induce hostile media perceptions, such as user-generated comments. Therefore, the following research questions and hypothesis guide this study:

RQ1: Do perceptions of story bias vary as a function of exposure to one-sided Facebook user comments and news story topic?
H1: Perceptions of reporting bias will vary as a function of exposure to both one-sided Facebook user comments and news story topic.
RQ2: Do perceptions of ethical reporting vary as a function of exposure to one-sided Facebook user comments and news story topic?

## Method

### Participants and procedures

Using a 2 (likeminded comments or disagreeable comments) × 2 (story topic of requiring vaccines to receive a monetary bonus or maintain employment) between-subjects experimental design, the current study recruited adult Facebook users to participate. Despite the abundant amount of user-generated data online (e.g., social media feedback on the pandemic), the use of alternative approaches is an important and necessary requirement for testing and advancing the hostile media bias phenomenon. Thus, an experimental research design allows for conditions to be created that provide a controlled and manipulated environment for Facebook news audiences. Furthermore, these conditions represented the various situations that were being experienced among employees in the U.S. at the time. With approval from the university-affiliated Institutional Review Board, the current study recruited adult Facebook users to participate using Dynata, a professional survey company contracted to collect a nationwide sample of U.S. adults (*N* = 1,274). The survey was fielded from May 9, 2021 to May 11, 2021. Dynata solicited potential respondents and asked them to voluntarily participate in exchange for credit to be used in their internal reward system. The online survey was hosted on a Qualtrics account associated with the university, and the data collection instrument did not collect any identifying information from participants.

### Stimulus

Upon agreeing to participate, respondents were asked several questions about their personal media use and psychological attributes, and then were randomly assigned to one of the two topics, either (a) offering monetary bonuses to employees who receive a COVID-19 vaccine; or (b) requiring employees to receive a COVID-19 vaccine to maintain their employment. After answering questions about their own opinions on the topic (i.e., either offering monetary bonuses to employees for receiving a COVID-19 vaccine or requiring employees to receive a COVID-19 vaccine to maintain their employment), participants were exposed to a Facebook news teaser—a post advertising a news story, from the Associated Press (A.P.), a news outlet audiences perceive as a balanced source (AllSides, 2021; Media Bias Fact Check, 2021).

Respondents were asked a series of questions that led them to reveal their own opinion on the randomly assigned topic before then being randomly assigned to a news teaser with comments that either agreed or disagreed with their stance on the topic. The Facebook post included a neutral headline and image. It featured three comments from users that were intentionally manipulated to represent likeminded (i.e., agreeable) or dissimilar (i.e., disagreeable) opinions on the issue as compared to the participant's opinion.

After reviewing the Facebook post and comments, each participant was directed to read a neutral news article on their assigned topic. The news stories that served as stimuli were created for this study by the authors, who have expertise in news writing and A.P. style. Stimuli content was created to present balanced content on either approach taken to increase the rate of COVID-19 vaccinations (i.e., the use of monetary incentives to employees who receive a vaccine or requiring employees to receive a vaccine to maintain their job). The only difference between the two stories was the topic, title, and in-text mentions of the respective approach used to increase vaccinations. Otherwise, the stimuli were standardized to maintain the story content across the two news stories. Once the news story was read, participants were then asked questions to assess their perceptions of story bias, reporting bias, and ethical reporting.

### Measures

#### Perceived story bias

Mirroring measurement from Fico et al. (2004), this item assesses the individual perception of news story bias. Measurement was accomplished with three items, which asked the participants to think about the news article they had just read and indicate how they felt the article provided information on the debated topic. The items included: (a) the amount of space and prominence the story gave to each side; (b) the strength of the arguments included for each side; and (c) the quality of the sources cited for each side. Responses were recorded using a 7-point Likert-type scale (1 = *strongly opposes requiring COVID-19 vaccines to _______* to 7 = *strongly favors requiring COVID-19 vaccines to ______*). The blank space was replaced with the randomly assigned topic condition (i.e., either receive a bonus or maintain employment). All items were found to be reliably related and were subsequently merged to form an index (α = 0.84; *M* = 4.38, *SD* = 1.12).

#### Reporting bias

Following previously validated measurement from Gunther and Schmitt (2004), this study examined multiple dimensions of perceived bias in reporting. Using items modified to fit the goals of this work, participants were asked three questions, including: (a) Do you feel that the news story was greatly biased against or in favor of your opinion about requiring vaccination to work?; (b) Do you feel that the writer of the news story was greatly biased against or in favor of your opinion about requiring vaccination to work?; and (c) Do you feel that the news outlet that published this story was greatly biased against or in favor of your opinion about requiring vaccination to work? Participant responses were collected with a 7-point Likert-type scale (1 = *strongly biased against my opinion _______* to 7 = *strongly biased in favor of my opinion _______*). The blank space was replaced with the randomly assigned topic condition (i.e., either receive a bonus or maintain employment). All items were found to be reliably related and were subsequently merged to form an index (α = 0.84; *M* = 4.04, *SD* = 1.16).

#### Perceived ethics

Perceptions of how ethically the news story was presented to readers were measured with the use of a single item. After reading the assigned article, participants were asked, “Did the article present _________ as unethical or ethical?” Responses were collected with a 7-point Likert-type scale (1 = *strongly unethical to require vaccines to _______* to 7 = *strongly ethical to require vaccines to ______*). The blank was replaced with the assigned topic (i.e., either receive a bonus or maintain employment) condition (*M* = 4.26, *SD* = 1.40).

#### Demographics

The average respondent was found to be 61.25 years old (*SD* = 15.70), and the slight majority were male (52.3%). Regarding educational status, the majority reported having completed between a 2-year and a 4-year college degree (*M* = 4.29, *SD* = 1.55). Most considered themselves to be between moderately conservative and independent in terms of their political ideology (1 = *very conservative* to 7 = *very liberal*) (*M* = 3.67, *SD* = 1.82). Concerning the race/ethnicity of respondents, the majority self-reported themselves as White/Caucasian (88.3%) and the estimated household income was found to range from $60,000 to under $70,000 (1 = <*$20,000* to 10 = *$100,000 or more*) (*M* = 6.20, *SD* = 3.22).

## Results

**Figure 1:**
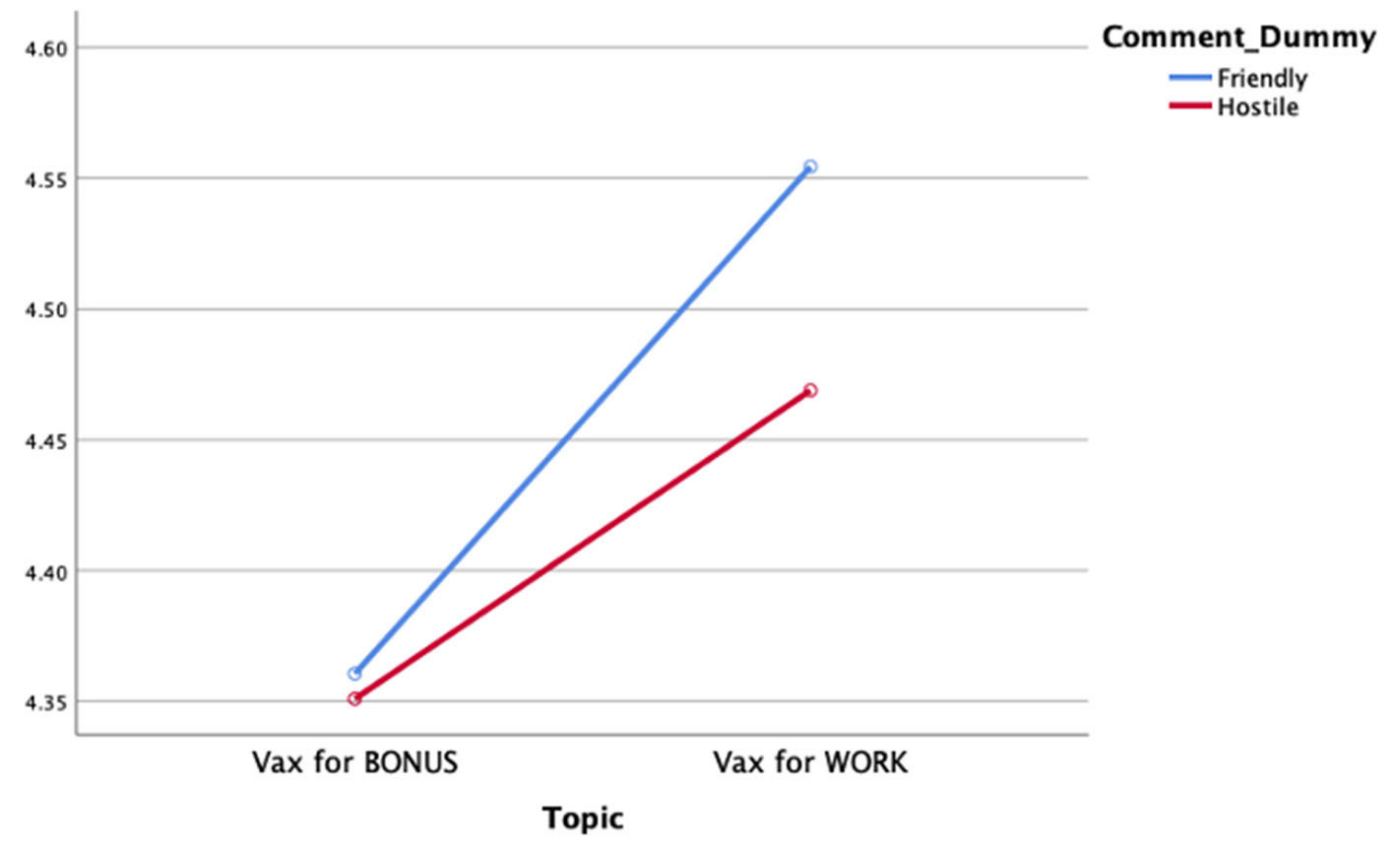
Perceived story bias by comment condition and story topic.

RQ1 asked whether perceptions of news story bias vary as a function of exposure to one-sided Facebook comments and news story topic. Data analysis revealed no main effect of exposure to one-sided Facebook comments on perceptions of story bias [*F*_(1,849)_ = 0.38, *p* = ns; ηp2 = 0.00]. However, as seen in Figure 1, data analysis demonstrated a significant main effect of news story topic on perceived bias [*F*_(1,849)_ = 5.12, *p* = 0.04; ηp2 = 0.01]. Review of associated means demonstrated that stories about requiring vaccinations to maintain employment are perceived to have a higher level of bias within the story (*M* = 4.51, *SD* = 1.16) than do stories about offering monetary incentives for receiving the vaccine (*M* = 4.35, *SD* = 1.08). No interaction effect was identified.

H1 predicted that perceptions of reporting bias will vary as a function of exposure to both one-sided Facebook user comments and news story topic. Data analysis revealed no main effect of exposure to one-sided Facebook comments on perceptions of story bias [*F*_(1,851)_ = 0.65, *p* = ns; ηp2 = 0.001]. Data analysis revealed a significant main effect of news story topic on reporting bias [*F*_(1,851)_ = 11.85, *p* = 0.001; ηp2 = 0.014]. Review of the associated means demonstrated that stories about requiring vaccinations to maintain employment are perceived to have a higher level of perceived reporting bias (*M* = 4.15, *SD* = 1.16) than do news stories about offering monetary incentives for receiving the vaccine (*M* = 3.87, *SD* = 1.14).

**Figure 2:**
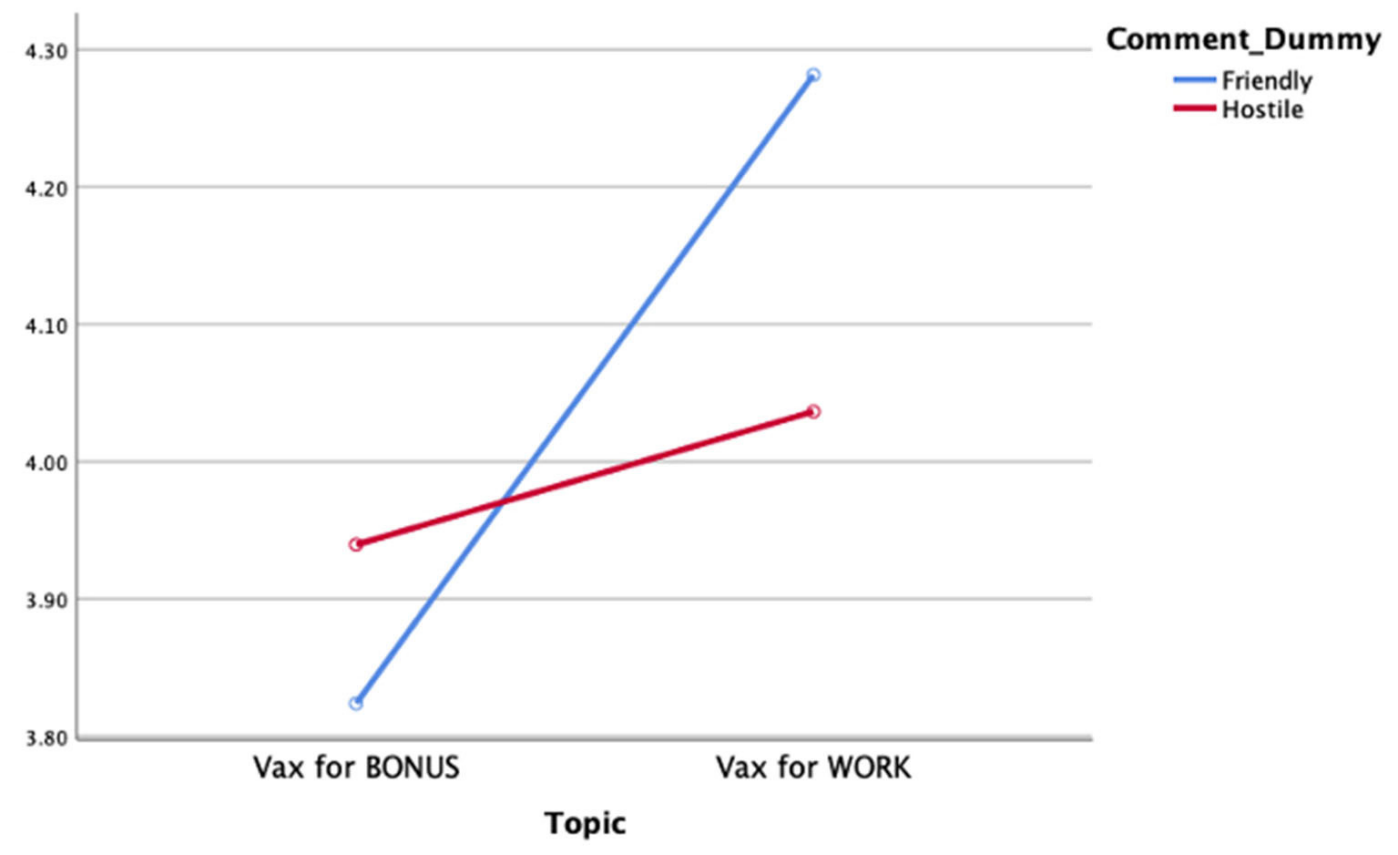
Reporting bias by comment condition and story topic.

Data analysis further revealed a significant interaction effect between exposure to one-sided Facebook comments and news story topic on perceptions of reporting bias among audiences [*F*_(1,851)_ = 5.03, *p* = 0.03; ηp2 = 0.01]. Review of the associated means indicate that exposure to likeminded comments before reading a story about requiring the vaccine to work result in higher perceptions of reporting bias (*M* = 4.25, *SD* = 1.11) than do viewing likeminded comments before the same story (*M* = 3.82, *SD* = 1.20), and when exposed to disagreeable Facebook user comments about either requiring vaccines for either employment or receipt of a financial incentive (*M* = 4.04, *SD* = 1.28; *M* = 3.94, *SD* = 1.06; respectively). Therefore, H1 was partially supported (see Figure 2).

RQ2 asked whether perceptions of ethical reporting vary as a function of exposure to one-sided Facebook user comments and news story topic. Data analysis revealed no main effect of exposure to one-sided Facebook comments on perceptions of ethical reporting [*F*_(1,853)_ = 0.86, *p* = *ns*; ηp2 = 0.001]. Data analysis further revealed a significant main effect of news story topic on perceived ethical reporting [*F*_(1,853)_ = 3.84, *p* = 0.04; ηp2 = 0.004]. Review of the associated means demonstrated that stories about requiring a COVID-19 vaccine to keep one's job are perceived to have a higher level of ethical reporting (*M* = 4.37, *SD* = 1.47) than were news stories about offering monetary incentives for vaccination (*M* = 4.18, *SD* = 1.33).

**Figure 3:**
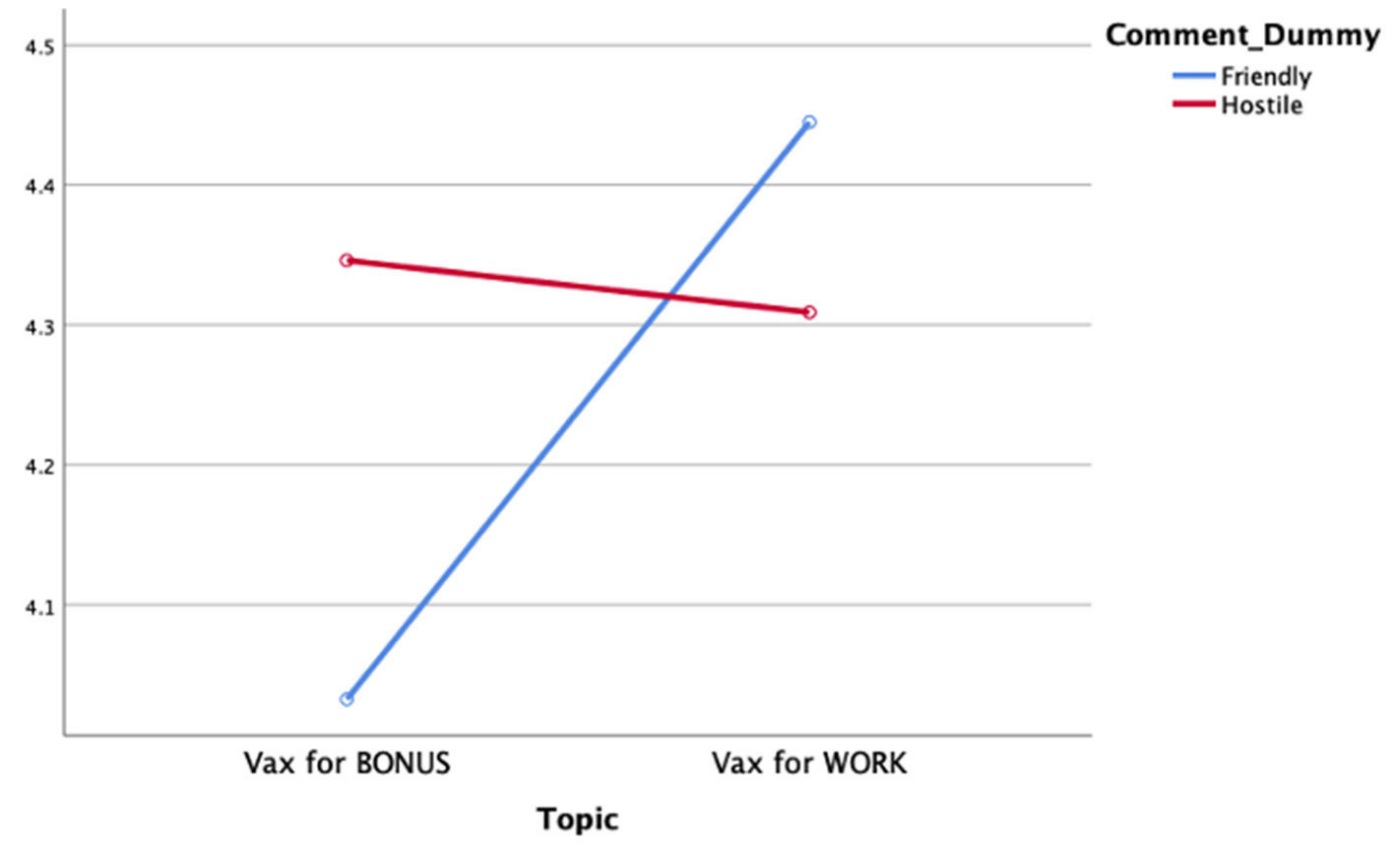
Perceptions of ethical reporting by comment condition and story topic.

As seen in Figure 3, data analysis further revealed a significant interaction effect between exposure to one-sided Facebook comments and news story topic on perceptions of reporting bias among audiences [*F*_(1,853)_ = 5.50, *p* = 0.02; ηp2 = 0.006]. Review of the associated means indicates that exposure to likeminded comments before reading a story about requiring the vaccine to maintain employment resulted in higher perceptions of ethical news reporting (*M* = 4.45, *SD* = 1.37) than when viewing likeminded comments before a story about requiring vaccinations to receive a monetary bonus (*M* = 4.03, *SD* = 1.35). However, perceptions of ethical reporting did not differ from other conditions when exposed to dissimilar Facebook comments for stories about requiring vaccines for either employment or receipt of a financial incentive (*M* = 4.31, *SD* = 1.55; *M* = 4.35, *SD* = 1.30; respectively).

## Discussion

The goal of the current study was to investigate how exposure to Facebook user comments can influence news audience perceptions of actual news content. Previous studies have investigated the influence of comments across controversial topics (Arceneaux et al., 2013; Kim, 2015; Gearhart et al., 2021). This project aimed to broaden the theoretical scope by testing whether different parts of the online news experience, like the inclusion of opinionated commentary before reading a neutral news story across different topics, influence one another rather than working in isolation. This work also systematically examines the influence of hostile media bias while removing the influence of partisanship and opinion strength, isolating the influence of comments and news story topic. Furthermore, this study uniquely assesses this variation across COVID-19 vaccine-related topics during the height of the pandemic to investigate responses related to tactics used to encourage vaccinations.

First, results demonstrate that only the more serious story topic directly influences perceptions of bias (i.e., vaccine necessary to keep employment). Specifically, Facebook users who were exposed to a news story about requiring vaccines to maintain one's employment found the news to strongly favor requiring COVID-19 vaccines, regardless of comment condition. However, the same was not true when the news story discussed the topic of requiring a vaccine to receive a financial incentive. Journalists reporting news in mainstream media tend to prioritize objective information, aiming to present a balanced story to audiences (Benham, 2020). In the case of the stories seen here, they were nearly identical, with the only variation concerning the type of requirement (i.e., either required to receive financial bonus or to maintain employment). Therefore, the serious nature of potentially losing one's employment appeared to have stronger implications for audiences. Audience perceptions of reporting bias differed based on the topic of the news story. Higher perceptions of reporting bias indicated that news audiences felt the story itself, the writer, and the news outlet that published the report were biased in favor of their own opinion on the topic (Gunther and Schmitt, 2004). D'Alessio (2003) found that news readers were more likely to view material as biased depending on the topic, especially when the topic was political in nature. In this case, both news stories were politically related. However, the topic with the stronger consequences, potentially facing punishment by losing one's job for refusing the COVID-19 vaccine, was found to induce perceptions of reporting bias.

Results also revealed that user-generated comments influence audience perceptions of news content. That is, exposure to likeminded Facebook comments encouraged perceptions of reporting bias when the news was about requiring COVID-19 vaccines to retain one's current employment. Identifying the influence of user comments on audience perceptions aligns with a growing body of research (e.g., Lee et al., 2018; Prochazka et al., 2018; Gearhart et al., 2020, 2021). More importantly, the identified interaction between exposure to opinion-congruent comments and news on the topic of employment requirements suggests that likeminded comments can enhance perceived bias in favor of one's opinion, especially when seen alongside intense story topics. While the intensity of a news topic may be viewed as a subjective construct often based on perceptions of personal topic importance (Tunney et al., 2021), the severity of losing one's job indicates the seriousness of the subject matter.

The topic of the news story was also found to influence perceptions of ethical reporting. Results regarding perceived reporting bias indicate news audiences found the news article to be more ethical when exposed to Facebook comments expressing compatible viewpoints on the topic of limiting employment to vaccinated individuals. This might be explained by the fact that audiences might perceive vaccine incentives as similar to offering a bribe, thus perceiving the news story about incentives as less ethical. Since this direction has not yet been further explored, the specialized focus on perceptions of ethical news reporting in this study encourages further hostile media bias research to include this concept. Future studies should further explicate the concept of ethical reporting as part of the hostile media phenomenon.

In summary, findings strongly suggest that the hostile media effect remains relevant in social networks. Overall, results align with previous research that has found perceptual biases linked to exposure to Facebook comments seen *before* viewing news content (Gearhart et al., 2020, 2021). This work may be limited by the quality of the sample, indicating that the Dynata sample may attract an older population of respondents. However, the sample notably features an audience of older Americans reminiscent of those adult U.S. Facebook users consuming news on the platform (Auxier and Anderson, 2021). While the current study supports existent findings regarding the influence of Facebook comments, this work uniquely identifies limitations to this influence that are dependent upon story topic. Specifically, the more intense topic of restricting employment only to individuals who have received the COVID-19 vaccine was found to interact with exposure to likeminded user comments. While this work indicates that the influence of social media comments may vary based on the intensity of news topic, future research directions should further explore how limiting the influence of comments seen before accessing news content to the most controversial issues. Therefore, results demonstrate the broad reach of perceptual biases and extend the theoretical scope. Practically speaking, implications of this work indicate that journalists should be aware of the impact that user-generated comments may have on perceptions of their reporting practices. Furthermore, social media users should consider how user-generated comments may hinder their ability to engage in meaningful dialogue and thoughtful exchanges through these platforms.

## Data availability statement

The raw data supporting the conclusions of this article will be made available by the authors, without undue reservation.

## Ethics statement

The studies involving human participants were reviewed and approved by Texas Tech University Institutional Review Board. Written informed consent for participation was not required for this study in accordance with the national legislation and the institutional requirements.

## Author contributions

SG led this grant-funded project alongside co-PI, IC, and led the methodological execution and data analysis. AM and SB supported the project through stimulus creation. All authors contributed to planning of the project, including theoretical scope, topic choice, literature review, and discussion. All authors contributed to the article and approved the submitted version.
